# Effect of Oil Content and Oil Addition Point on the Extrusion Processing of Wheat Gluten-Based Meat Analogues

**DOI:** 10.3390/foods10040697

**Published:** 2021-03-25

**Authors:** Christina Kendler, Arvid Duchardt, Heike P. Karbstein, M. Azad Emin

**Affiliations:** Institute of Process Engineering in Life Sciences, Chair of Food Process Engineering, Karlsruhe Institute of Technology, 76131 Karlsruhe, Germany; christina.kendler@kit.edu (C.K.); arvid.d@gmx.de (A.D.); heike.karbstein@kit.edu (H.P.K.)

**Keywords:** high-moisture extrusion, meat analog, MCT oil, wheat gluten, rheological properties, anisotropic structure, oil droplet distribution, CLSM

## Abstract

High-moisture extrusion is a common process to impart an anisotropic, meat-like structure to plant proteins, such as wheat gluten. The addition of oil during the process promises to enhance the sensory properties of the meat analogs. In this study, the influence of oil on extrusion-relevant parameters as well as the structure-related characteristics of extruded wheat gluten was investigated. Oil was added directly to the extruder at different contents (0, 2, 4, 6%) and addition points (front/end of the extruder barrel). Process conditions, complex viscosity, Young’s modulus and oil phase morphology were determined as a function of oil content and oil addition point. With increasing oil content, material temperature, die pressure, and complex viscosity decreased. The addition of oil at the end of the extruder barrel reduced this effect compared to the addition of oil in the front part of the extruder. It was observed that the extrudate’s tensile strength is a function of material temperature, resulting in an increase in tensile strength with increasing material temperature. The oil was dispersed in the gluten matrix as small droplets with irregular shape. As the oil content increased, the size of the oil droplets increased, while the addition of oil at the end of the extruder resulted in a decrease in droplet size.

## 1. Introduction

Health, ecological, and ethical reasons motivate consumers to change their eating habits to a mainly plant-based but, nevertheless, balanced diet. However, many consumers are not willing to compromise on the sensory properties of animal products. Therefore, the demand for protein-rich products on a plant basis with an appealing, meat-like texture is constantly increasing [1,2,3]. Plant proteins can be texturized to generate a fibrous and anisotropic structure that resembles the characteristic properties of meat. A common process to create these is high-moisture extrusion with an attached cooling die. During the extrusion process, the plant proteins are mixed with water, heated, sheared, and forced through the cooling die, resulting in the formation of an anisotropic structure [4,5,6,7,8,9,10,11,12,13,14,15].

The consumer’s expectations for the sensory properties of meat analogues are the same as the expectations for the typical sensory properties of meat. The most important sensory properties of meat are its flavor and texture, which include, in particular, the tenderness and juiciness of the meat [1,16,17]. In meat, these characteristic properties are generated by the composition of muscle fibers and intramuscular fat. Depending on origin and type, meat can contain up to 30% fat [18,19,20,21]. In meat analogues, the texture of the meat muscle fibers is simulated by the formation of anisotropic structures. However, these structures alone do not sufficiently reflect the texture of meat, especially in terms of juiciness [2,22,23]. As with meat, it can be assumed that these sensory properties can be improved by adding fat or oil to the plant-based meat analogues. Nevertheless, it must be considered that the addition of oil not only influences the juiciness of the product, but also has an impact on the extrusion process conditions, rheological properties and eventually on the formation of anisotropic structures.

During the high-moisture extrusion of meat analogues, proteins are mixed with water, heated, and sheared by the rotation of the screws. In this screw section, the proteins undergo a number of protein–protein interactions, resulting in a change of the rheological properties of the protein matrix. During extrusion processing, the protein matrix forms a multiphase system with an inhomogeneous water distribution. In the cooling die, this multiphase protein matrix is deformed and aligned along the flow direction, creating an anisotropic product structure [11]. The review paper by Cornet et al. [24] provides a detailed overview of the possible mechanisms found in literature that lead to the formation of an anisotropic structure in meat analogues. For its formation, the flow characteristics and the rheological properties in the die section play a crucial role [25,26]. The importance of controlling rheological properties in extrusion processing due to their great impact on process conditions and the formation of final product properties has been reported for various low-moisture [27,28,29] as well as high-moisture extrusion applications [13,26,30].

For wheat gluten, which was used as a model protein system in this paper, it is known that the gluten molecules are highly reactive and undergo polymerization reactions due to the thermomechanical energy input in the extrusion process. It has been reported that cross-linking via disulfide bonds plays an important role in the formation of a three-dimensional protein network. Several studies have focused in detail on the intermolecular and intramolecular protein interactions formed as a function of extrusion process conditions [9,31,32,33,34]. Emin et al. [35] investigated the relationship between the reaction behavior of gluten molecules and the resulting rheological properties in a closed cavity rheometer under extrusion-like conditions. In this study, it was reported that the polymerization reactions of gluten are strongly temperature and shear stress dependent, whereby an increase in polymerization reactions led to a remarkable increase in material viscosity.

In order to gain a mechanistic understanding of the complex interrelationships in the extrusion process, it is necessary to consider and discuss how the addition of oil influences the process conditions, polymerization reactions of gluten, rheological properties and formation of anisotropic product structure [36]. Only very few studies on the addition of oil in the high-moisture extrusion of meat analogues are found in literature [31,32,37]. Jia et al. [31] investigated the influence of plant oil on the formation of protein–protein interactions during the extrusion of wheat gluten. They extruded wheat gluten premixed with different amounts of oil blends containing peanut oil, soybean oil and rapeseed oil. Thereby, they observed that the addition of up to 8% oil led to a decrease in the free sulfhydryl groups of the gluten, suggesting that the polymerization of the gluten via disulfide bonds is enhanced by the addition of oil. However, in this study, no correlations between oil addition, extrusion conditions and resulting material and product properties were provided. Gwiazda et al. [37] investigated the influence of oil on the formation of anisotropic structures for meat analogues on soy protein basis. The authors observed that oil is dispersed into the protein matrix as small, spherical droplets. Moreover, they reported that oil inhibits the formation of an anisotropic structure, leading to a decrease in tensile strength. This study provides basic knowledge on how oil affects the product properties of extruded meat analogues.

The objective of this study is, therefore, to investigate the influence of oil on the high-moisture extrusion of wheat gluten as a model protein system in more detail. It will be determined how oil affects the process conditions and rheological properties as a function of the oil content and the oil addition point. This will help to provide a better understanding of how oil influences the formation of anisotropic structures. Moreover, we will show how the added oil is dispersed in the gluten matrix and to what extent the process parameters and rheological properties influence the morphology of this dispersed phase.

## 2. Materials and Methods

### 2.1. Materials

Vital wheat gluten was supplied by Kröner Stärke (Ibbenbüren, Germany). According to the manufacturer’s information, the vital wheat gluten contains max. 8% moisture and 83% protein on a dry matter basis. Throughout this paper, it is referred to as “gluten”. Medium-chain triglycerides oil (MCT oil, WITARIX^®^60/40) was purchased from IOI Oleo GmbH (Hamburg, Germany) and is referred to as “oil” throughout this paper. In order to visualize the oil under confocal laser scanning microscopy (CLSM), the lipophilic fluorescent dye nile red, purchased from Carl Roth (Karlsruhe, Germany), was dissolved in the oil in a concentration of 0.02 g/L.

### 2.2. Extrusion Processing

**Figure 1:**
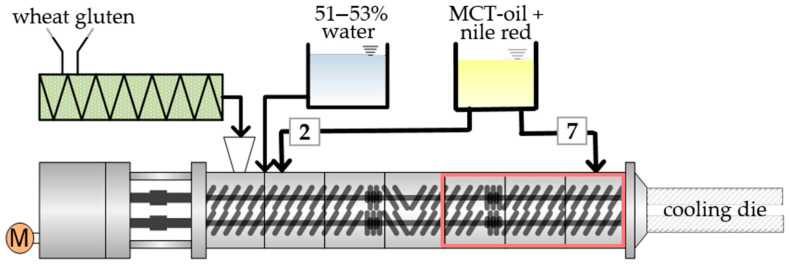
Schematic illustration of experimental setup for extrusion trials. Variable process parameters were the oil content (0, 2, 4, 6%), oil addition point (position 2 and 7), screw speed (200, 400, 800 rpm) and barrel temperature in segments 5–7 (120 or 140 °C).

The extrusion trials were performed in a co-rotating twin-screw extruder ZSK 26 Mc (Coperion, Stuttgart, Germany) with a screw diameter of 25.5 mm and a length to diameter ratio of 29. The experimental setup is depicted in Figure 1. The extruder barrel consists of seven barrel segments that can be heated separately except for the first segment. Attached to the barrel section was a rectangular cooling die with the dimensions 380 × 30 × 9 mm^3^ (l × w × h). The temperature of the cooling die was set to 40 °C with a refrigeration unit (Presto Plus LH 47, Julabo GmbH, Seelbach; Germany). For all experiments, the same screw configuration consisting of forward and reverse transport elements and two kneading blocks was used. The gluten was fed into the first barrel segment by a gravimetrically controlled feeder DDW-DDSR40 (Brabender, Duisburg, Germany). The water was pumped with a piston-membrane pump (model KM 251, Alldos, Pfinztal, Germany) into the second barrel segment. The oil stained with nile red was added by a HPLC-pump HD 2-200 (Besta-Technik GmbH, Wilhelmsfeld, Germany) at two different addition points, either in the second barrel segment after the water addition, which is referred to as “position 2” or in the last barrel segment before the attached cooling die, which is referred to as “position 7”. The extrusion experiments were performed at different barrel temperatures, screw speeds (200, 400, 800 rpm) and oil contents (0, 2, 4, 6%). The barrel was heated with a temperature profile of 40, 60, and 80 °C for the barrel segments 2, 3 and 4, and the last three segments (5–7) were set to either 120 or 140 °C, respectively. With increasing oil content, the water–gluten ratio was kept constant at 1:1. This results in water contents between 51 and 53%. The total mass flow rate was constantly adjusted to 10 kg/h. To characterize the process conditions, material temperature and process pressure were measured at the end of the extruder barrel, and torque was detected. After the process conditions were stable for at least three minutes, extrudate samples were taken and then frozen in airtight plastic bags at −18 °C until further examination.

### 2.3. Rheological Measurements

**Figure 2:**
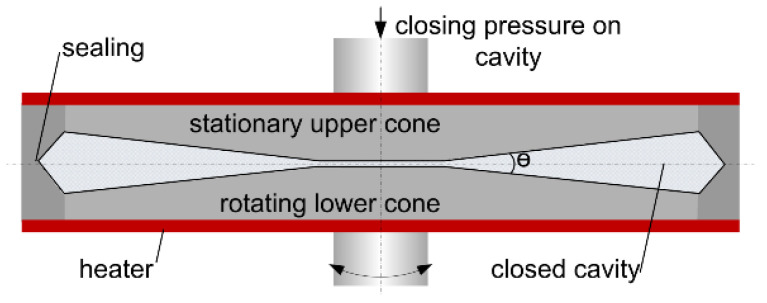
Schematic illustration of the closed cavity rheometer (CCR) used for rheological measurements.

Due to the water loss after extrusion, it is necessary to adjust the moisture content of all samples before the rheological measurements. Therefore, the extrudate samples were pre-dried, ground to a particle size < 500 µm, dried to mass consistency and rehydrated to the original extrusion moisture content in a Thermomix (Vorwerk, Wuppertal, Germany). In addition to the rehydrated extrudate samples, gluten doughs were prepared by mixing the untreated wheat gluten powder with oil and water. To ensure homogeneous water distribution and hydration, all samples and doughs were stored under vacuum at 4 °C for at least 12 h before the measurements. The complex viscosity of the rehydrated samples and the untreated gluten doughs was determined by an oscillatory closed cavity rheometer (RPA elite, TA Instruments, New Castle, DE, USA), which is shown schematically in Figure 2.

The function of the closed cavity rheometer is previously described in more detail [35,38,39]. For the measurements, 5.5 g of the sample was placed between the two cones of the cavity. The cavity was sealed and pressurized to prevent water loss during the measurements. Both cones are temperature controlled and the lower cone oscillates with a defined frequency and strain amplitude. From the resulting torque response, rheological properties such as complex viscosity can be calculated. To evaluate the influence of the extrusion process on the material properties, the complex viscosity η* was determined as a function of the angular frequency. For this, frequency sweeps from 1 to 50 Hz at a constant strain amplitude of 5% (within the linear viscoelastic region) were performed.

### 2.4. Visualization of Anisotropic Structure

The anisotropic structure of the extrudate samples was visually assessed immediately after their processing. Therefore, the extrudates were cut along the flow direction using a blade and opened relatively to the flow direction. Furthermore, the anisotropic structure was visualized by X-ray measurements in a micro-computed tomograph (micro-CT). To prepare the samples for micro-CT, they were cut into pieces and freeze-dried. Drying was performed at −80 °C and 50 mbar in an Alpha 1–4 LDplus laboratory freeze dryer (Martin Christ Gefriertrocknungsanlagen GmbH, Osterode, Germany). The freeze-dried samples were analyzed in the micro-CT Xradia 520 (Carl Zeiss Microscopy GmbH, Oberkochen, Germany). Therefore, the samples were fixated in a sample holder, which was placed between the radiation source and the detector. For each measurement, the samples are rotated 360° in the X-ray beam between the radiation source and the detector. Digital image processing software (ImageJ software version 1.50d, National Institutes of Health, Bethesda, MD, USA) was used for noise filtering and segmentation.

### 2.5. Analysis of Texture Properties

**Figure 3:**
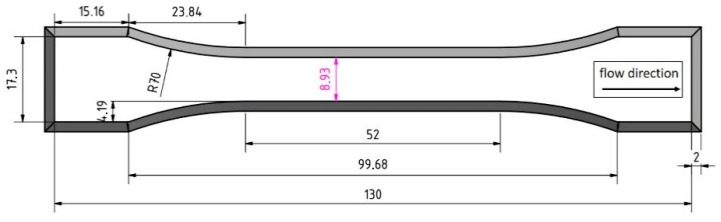
Dimensions of the bone-shaped specimen used for tensile strength tests taken from Pietsch et al. [33].

To evaluate the texture properties of the extrudate samples, tensile strength analyses were performed with a texture analyzer (Z2.5 TS, Zwick Roell, Ulm, Germany). Therefore, a probe with the dimensions shown in Figure 3 was cut from the extrudate sample. The bone-shaped probe was fixed to the testing geometry type 8201 (Zwick Roell, Ulm, Germany) on both ends and stretched at a speed of 1 mm/s until breakup. All tensile tests were performed at least seven times per sample.

During the measurement, the stress–strain diagram was recorded. From the linear region of the stress–strain diagram, the Young’s modulus (E) was calculated using Equation (1), as described by [38,40].

(1) $$E\;=\;\frac{F}{A\;\times \;\varepsilon }$$

*F* is the measured force, *A* is the cross-sectional area of the specimen and *ε* is the strain, which was calculated as shown in Equation (2).

(2) $$\varepsilon \;=\;\frac{\Delta l}{l_{0}}$$

*l*_0_ is the original length of the specimen, whereas Δ*l* is the change in the original length of the specimen caused by the applied stress.

### 2.6. Oil Droplet Size Measurements

The oil droplets in the extrudate samples were visualized by confocal laser scanning microscopy (CLSM). For this, the oil was stained with the fluorescent dye nile red in a concentration of 0.02 g/L before extrusion processing, the protein matrix is autofluorescent. To prepare the samples for CLSM, the samples were cut with a CM 3050 cryo-microtome (Leica Biosystems GmbH, Nussloch, Germany). For this purpose, small pieces of the extrudate samples (20 × 9 × 9 mm) were embedded in a cutting medium (FSC 22 Blue Frozen Section media, Leica Biosystems GmbH, Nussloch, Germany) and fixed on the sample holder in the cutting chamber of the cryo-microtome at −16 °C. In the cryo-microtome, the samples were cut into 40 µm thick slices. Immediately after cutting, the slices were placed on microscope slides and dried at 55 °C. After drying, the slices were fixated on the slides with Mowiol (Carl Roth, Karlsruhe, Germany) and covered with a cover slide.

The samples fixed on the microscope slide were analyzed with the LSM 510 META microscope (Carl Zeiss Microscope Systems, Jena, Germany) with a Plan-Apochromat 63×/1.4 oil DIC immersion objective. The samples were excited with an argon laser at a wavelength of 488 nm. The emitted light was filtered with two different channels to separate the signal of the nile-red-stained oil (530–600 nm) from the protein signal (420–515 nm). At least 10 images were taken from each sample, so that at least 1058 droplets per sample were evaluated by image processing. Digital image processing software (ImageJ software, National Institutes of Health, USA) was used to analyze the oil droplet area. An area-weighted cumulative droplet size distribution (Q_2_) was plotted from the determined oil droplet areas.

## 3. Results and Discussion

### 3.1. Influence of Oil Addition on the Extrusion Process Conditions

**Figure 4:**
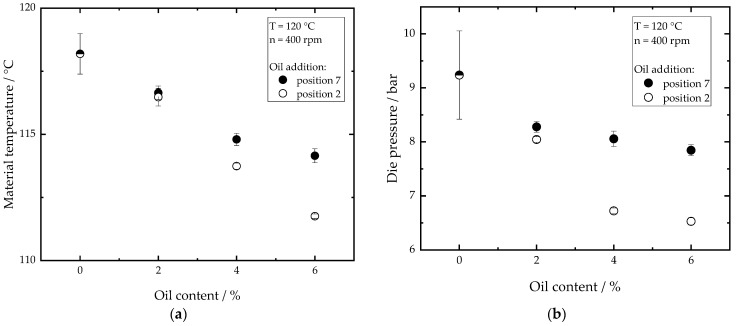
Effect of oil content on the (**a**) material temperature and (**b**) die pressure for oil addition point at position 2 and 7. Total mass flow was 10 kg/h and water content was 51–53% for all samples.

To characterize the influence of the oil on the process conditions, material temperature, die pressure and torque were measured. Figure 4a shows the influence of the oil content on the material temperature. With increasing oil content, the material temperature measured at the end of the screw section decreases. This effect can be observed for both oil addition points. The temperature drop is up to 7 K when oil is added at position 2. The decrease in material temperature with increasing oil content can be explained by a change in rheological properties, since the addition of oil is expected to reduce the viscosity of the material [41,42,43]. Moreover, oil is known to lubricate the interacting particles in the material and the particles that rub against the screw and barrel surfaces [44,45]. The reduction in viscosity, in combination with the lubricating effect, reduces shear stresses in the extruder and viscous energy dissipation, which leads to a decrease in material temperature and extruder torque.

As depicted in Figure 4b, the die pressure also decreases with increasing oil content. The die pressure in the extrusion process is described as a function of the material viscosity [10]. This decrease in die pressure implies a decrease in viscosity. The material viscosity is a function of die geometry, flow rate, material temperature and composition [46]. As the geometry and the flow rate were kept constant, the change in the die pressure can be related to material temperature and composition. According to the Arrhenius equation, a decrease in material temperature is expected to lead to a higher viscosity and thus to a higher die pressure, unless the material structure changes. Despite this temperature effect, the results in Figure 4b show a decrease in the die pressure, which can therefore be directly related to lower material viscosity. Therefore, we suggest that the addition of oil leads, on the one hand, to a direct reduction in viscosity. This can be attributed to a dilution of the highly viscous gluten matrix by reducing the polymer concentration per unit volume as well as to an increased molecular mobility of the polymer molecules. On the other hand, the addition of oil leads to an indirect reduction in viscosity by lowering the rate of polymerization reactions of the gluten molecules. This is expected to happen due to the lower initial viscosity of the material and the possible lubricating effect of the oil, resulting in a lower thermomechanical energy input, as shown in Figure 4. For further discussion, this combined effect is referred to as ‘*amplified oil effect*’. It is apparent that adding oil at position 7 has less impact on the temperature and pressure drop than adding oil at position 2. This shows that the *amplified oil effect* can be reduced by adding oil at the end of the screw section (position 7), as this allows the gluten polymerization and the viscosity build-up without the presence of oil until the position 7.

**Figure 5:**
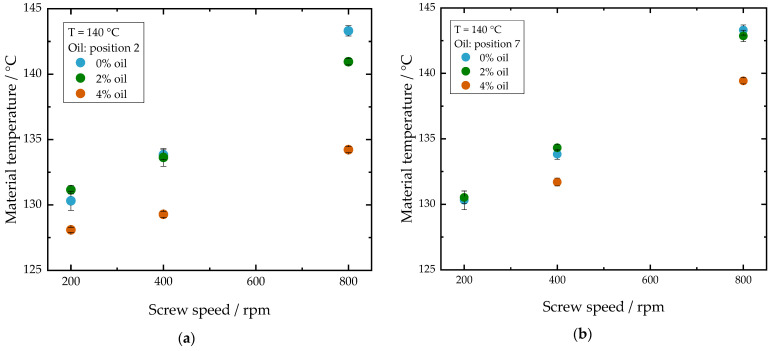
Influence of screw speed on material temperature for oil addition at (**a**) position 2 and (**b**) position 7 for different oil contents. Total mass flow was 10 kg/h and water content was 51–53% for all samples.

Figure 5 shows the influence of screw speed on material temperature for various oil contents and the oil addition at (a) position 2 and at (b) position 7. Both diagrams show that with increasing screw speed the material temperature increases for all oil contents. At higher screw speeds, more mechanical energy is introduced into the material and thus the temperature increases. As depicted in Figure 5a, at low screw speeds (200 rpm) the material temperatures between the different oil contents (0, 2, 4%) differ only by up to 4 K. At higher screw speeds, however, the differences between the different oil contents are much more pronounced. At 800 rpm, an addition of 4% oil at position 2 causes the material temperature to drop by almost 10 K. The greater influence of the oil on the process conditions at 800 rpm compared to 200 rpm can be explained by the fact that viscous dissipation plays a more significant role under these conditions. If the oil is added at the end of the extruder (Figure 5b), the addition of 4% oil at a screw speed of 800 rpm results in a temperature decrease of only 4 K. These results support the assumption that adding oil at position 7 reduces the *amplified oil effect* on the process.

### 3.2. Influence of Oil Addition on the Rheological Properties

**Figure 6:**
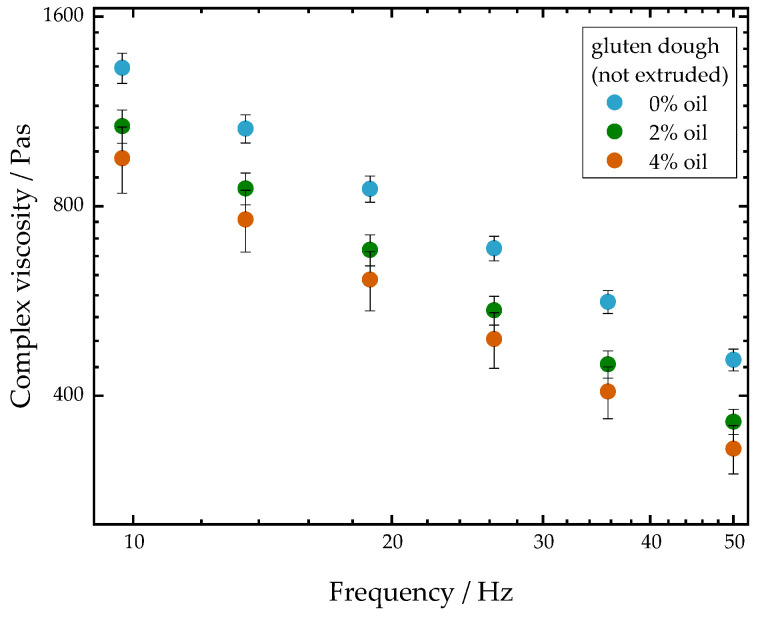
Influence of 0% oil (blue), 2% oil (green) and 4% oil (orange) on the complex viscosity of untreated gluten doughs (not extruded). Water content was 38–40%.

In order to determine the influence of the oil content on the rheological properties, the complex viscosity of the extrudate samples as well as of untreated gluten doughs (not extruded) was measured as a function of frequency. Figure 6 depicts the complex viscosity of untreated gluten doughs with different oil contents. The results show that the complex viscosity decreases with increasing frequency for all doughs. This is due to the typical shear-thinning behavior of highly concentrated biopolymers [46]. Furthermore, it can be seen from Figure 6 that the complex viscosity decreases with increasing oil content. These results are in agreement with the findings of Fu et al. [47], who investigated the effect of fat addition on the rheological properties of wheat flour dough. They also observed that viscosity decreased with increasing fat content. They suggested that oil does not lead to a decrease in viscosity by a simple dilution effect, but oil acts as a plasticizer by increasing the molecular mobility of wheat flour.

**Figure 7:**
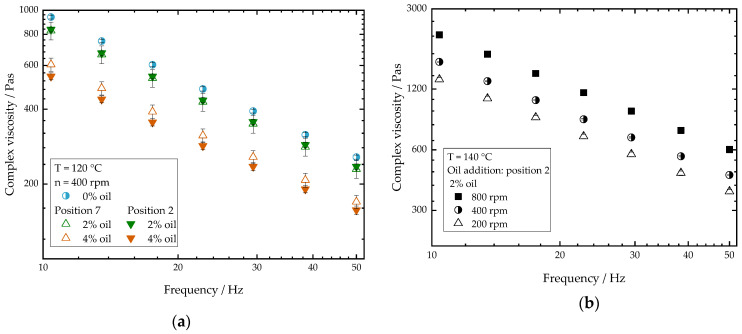
Complex viscosity as a function of the frequency. (**a**) Influence of different oil contents on the complex viscosity as a function of the oil addition point. Position 7 corresponds to open symbols; position 2 corresponds to filled symbols. (**b**) Effect of screw speed on the complex viscosity. Total mass flow was 10 kg/h and water content was 51–53% for all samples.

In Figure 7, the complex viscosity is depicted for different extrudate samples as a function of the frequency. The results in Figure 7a confirm that the oil addition, regardless of the addition point, decreases the material viscosity as expected. The addition point of the oil seems to have no effect at oil concentrations of 2%. At oil contents of 4%, however, it can be seen that adding oil at position 7 leads to higher complex viscosities than adding oil at position 2. These results are consistent with the observations on the influence of oil on process conditions (see the Section 3.1). This implies that, if oil is added at the front of the process, the polymerization of the gluten is strongly influenced by the oil, which is reflected in a lower complex viscosity. However, if the oil is added in position 7, the polymerization is expected to be less influenced by the oil, so that the viscosity build-up is higher.

In Figure 7b, the influence of the screw speed on the complex viscosity is depicted. As the screw speed increases, the complex viscosity increases considerably. Shown are the results for samples with 2% oil; for samples without oil, the same trends are seen. The results are consistent with observations on the effect of screw speed on material temperatures. Increasing the screw speed, on the one hand, reduces the residence time of the material in the extruder barrel, which might reduce the time for the gluten molecules to polymerize [48]. On the other hand, as screw speed increases more thermomechanical energy is introduced into the matrix. As the gluten molecules experience higher thermomechanical treatment, polymerization reactions are assumed to be enhanced, which leads to an increase in complex viscosity. These results are consistent with the results of Pietsch et al. [33], who reported an increase in the polymerization degree of gluten with increasing screw speed.

### 3.3. Influence of Oil on the Formation of Anisotropic Product Structure

The formation of the anisotropic structure of the extrudates was visually assessed immediately after extrusion processing. Table 1 shows samples with different oil contents where the oil was added at two different addition points. No remarkable differences in anisotropic structure formation between oil addition at position 2 and 7 can be seen in the images of the teared extrudate samples. However, when comparing the samples with different oil contents, a change in the anisotropic structure is visible. While hardly any differences in structure can be seen between the samples with 0% and 2% oil, the samples with 4% oil show a less pronounced anisotropic structure. The 4% oil samples look more dough-like and the orientation of the flow profile is less pronounced compared to the 0% and 2% oil samples. That the addition of oil leads to a loss of anisotropic structure formation was also reported by Gwiazda et al. [37]. They analyzed light micrographs of extruded soybean meal premixed with 0–15% soybean oil and observed that with increasing oil content the lengthwise orientation of the so called ‘fibers’ decreased.

The micro-CT images of the same samples are depicted in Table 2. Similar to the images in Table 1, a loss of the anisotropic structure with increasing oil content can be seen, particularly for the samples with added oil at position 2. Moreover, small differences between position 2 and 7 can be noticed in the micro-CT images. For example, the 4% oil sample with oil addition at position 2 shows hardly any texture, while the 4% sample with oil addition at position 7 still shows an anisotropic structure oriented along the flow direction in the cooling die.

Since micro-CT imaging is a non-invasive method for assessing three-dimensional network structures, it is expected that small differences in anisotropic structure can be visualized more precisely with this non-invasive method than with the invasive method of tearing the extrudates. It is known from the literature that, in high-moisture extrusion, the anisotropic product structure is formed by the multiphase protein matrix being deformed by shear stresses in the cooling die [11,24]. However, it is possible that in our case the added oil leads to wall slip regardless of the addition point and the rheological properties. As a result, the shear stresses in the cooling die, which are necessary to deform the matrix in the flow direction, would greatly decrease. This would explain why the samples show only little differences in their anisotropic structure for both addition points.

Increasing the screw speed leads to a more pronounced anisotropic structure. This trend can be seen in Table 3 for samples with 0% and 4% oil, which were teared immediately after extrusion processing. For all samples, oil was added at position 2 in the extruder. In Table 4, the micro-CT images of the same samples are depicted. These images confirm the observation that increasing the screw speed results in more pronounced anisotropic structures for samples with both 0% and 4% oil. As the screw speed increases, the gluten molecules experience more thermomechanical stress and thus the material temperature increases. This leads to an increase in polymerization reactions and with this to higher viscosities, as shown in Figure 7b. Higher material viscosity results in higher deformation stresses in the cooling die, which are necessary for the anisotropic structure formation [33].

### 3.4. Influence of Oil Addition on the Mechanical Properties

**Figure 8:**
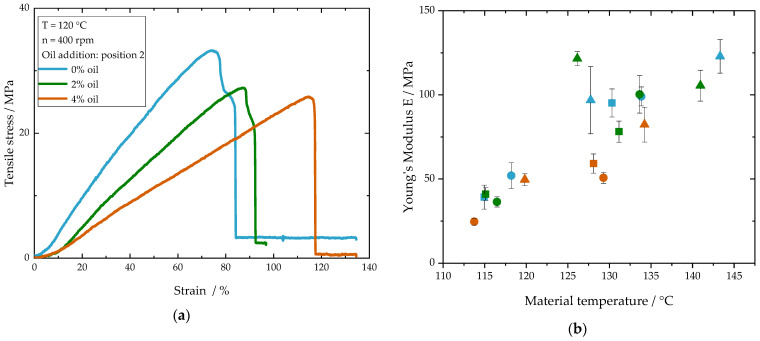
(**a**) Stress–strain-diagrams for samples with 0%, 2% and 4% oil. (**b**) Young’s modulus as a function of the material temperature for samples with 0% oil (blue), 2% oil (green) and 4% oil (orange) extruded with 200 rpm (■), 400 rpm (●) and 800 rpm (▲) at different barrel temperatures (120 and 140 °C). Oil was added at position 2. Total mass flow was 10 kg/h and water content was 51–53% for all samples.

In order to better understand the oil influence on the product properties, the mechanical properties were determined by tensile tests. Figure 8a shows individual stress–strain diagrams for samples with different oil contents where the oil was added at position 2. It can be seen that the curves initially have a linear slope until the tensile stress reaches its maximum and the specimen breaks. 

For the samples shown in Figure 8a, the mean Young’s modulus is listed in Table 5. The oil content is shown to have an impact on the mechanical properties of samples where oil was added at position 2. The Young’s modulus decreases by about half with the addition of 4% oil. When oil was added at position 2, the polymerization of the gluten is expected to be reduced due to the *amplified oil effect,* resulting in a weaker protein network. For this, the Young’s modulus as a mechanical property decreases with increasing oil content. 

The Young’s modulus for the samples where oil was added at position 7 are also listed in Table 5. In these samples, a strong protein network has already formed before the oil was added. This explains that the Young’s modulus of these samples shows no remarkable changes with increasing oil content. It can be concluded that the mechanical properties are a function of the polymerization degree of the matrix. The polymerization degree itself is strongly dependent on the process conditions. Pietsch et al. [33] showed that with increasing material temperature the polymerization degree of gluten and thus the Young’s modulus increased. To show the dependency between process conditions and mechanical properties, the Young’s modulus is plotted against the material temperature (Figure 8b). In the diagram, samples with different oil contents, screw speeds and barrel temperatures are depicted. The diagram shows that the Young’s modulus increases with increasing material temperature. The results indicate that the process conditions, which are a function of the oil content as well as the oil addition point, are the major factors influencing the formation of the mechanical properties.

### 3.5. Morphology of the Oil Phase in the Protein Matrix

**Figure 9:**
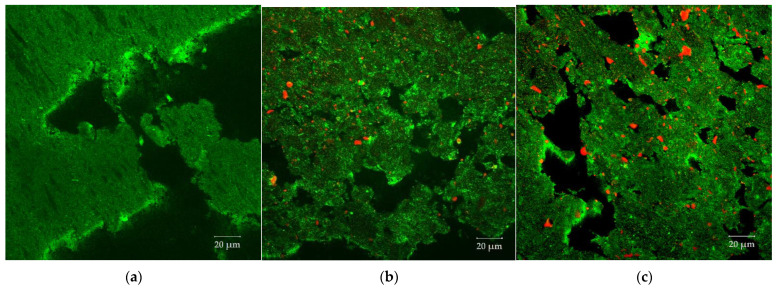
Morphology of the dispersed oil phase (red) in the protein matrix (green) for different oil contents. Confocal laser scanning microscopy (CLSM) images of samples with (**a**) 0%, (**b**) 2% and (**c**) 4% oil that was added at position 2. The barrel temperature was 120 °C and screw speed was 400 rpm.

The oil phase in the protein matrix was visualized with confocal laser scanning microscopy. In Figure 9, CLSM images of samples with three different oil contents where oil was added at position 2 are depicted. In the images, the oil appears red and the protein matrix green. It can be seen that the oil is dispersed into small droplets within the protein matrix. The same observation was made by Gwiazda et al. [37]. They reported that, after extrusion processing, soybean oil is dispersed as small spherical droplets in a mesh-like soy protein matrix. As can be seen from the CLSM images in Figure 9, the number of oil droplets, as well as the droplet size, increases with increasing oil content. That the droplet size increases with increasing oil content has already been observed by Emin et al. [49] for the encapsulation of oil droplets in starch matrices. Due to the increased oil content, the number of droplets in the matrix, and therefore the collision frequency, increases, which may enhance the probability of coalescence [50]. Some of the dispersed oil droplets do not have a round shape. The oil droplets are entrapped in a highly viscous protein matrix. This could explain why the droplets are fixated in the matrix in irregular, deformed shapes.

**Figure 10:**
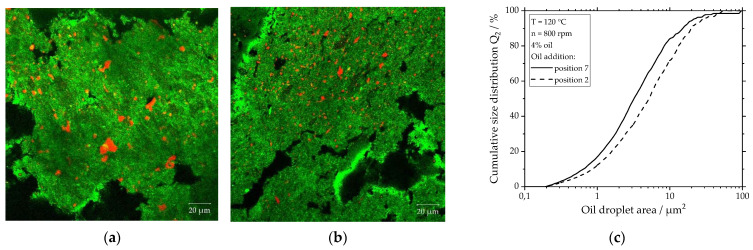
Influence of the oil addition point on the morphology and droplet size of the dispersed oil phase (red) in the protein matrix (green). CLSM images of samples with 4% oil that was added at (**a**) position 2 and (**b**) position 7. (**c**) Oil droplet size distribution for the samples depicted in (**a**) and (**b**). The barrel temperature was 120 °C and screw speed was 800 rpm.

To evaluate the influence of the oil addition point on the oil droplet size, CLSM images from samples with 4% oil where oil was added at two different addition points are depicted in Figure 10a,b. For (a) the oil was added at position 2, while for (b) the oil was added at position 7. Comparing the pictures from (a) and (b), it is evident that the oil droplets of the sample with oil addition at position 2 are larger than those of the sample with oil addition at position 7. For both samples, the droplet size distributions are depicted in Figure 10c. Because of the irregular droplet shape, the cumulative size distribution (Q_2_) is referred to the droplet area and not to the droplet diameter. Both curves show a relatively wide, mostly monomodal distribution with droplet areas ranging from 0.3 up to 100 µm. The curve of samples with oil addition at position 7 is shifted to the left compared to the curve with oil addition at position 2. This indicates that adding oil at position 7 leads to the formation of smaller oil droplets compared to adding oil at position 2. Thus, the results of the cumulative size distributions evaluated via image processing are consistent with the findings observed on the CLSM images. 

The viscosity of the matrix is expected to play a major role in droplet breakup as the mechanical stresses acting on the droplets are a function of the viscosity [51]. If oil is added at position 2, the material viscosity remains lower compared to the oil addition at position 7, as discussed in previous sections (see Figure 7a). The higher viscosity of the protein matrix at position 7 is therefore expected to lead to higher mechanical stresses and thus to higher oil droplet deformation and breakup. This explains the fact that smaller droplets are found when oil is added at position 7 instead of position 2. For the dispersive mixing of oil into a plasticized starch matrix, Emin at al. [49] also observed that smaller oil droplets were formed as the matrix viscosity increased.

**Figure 11:**
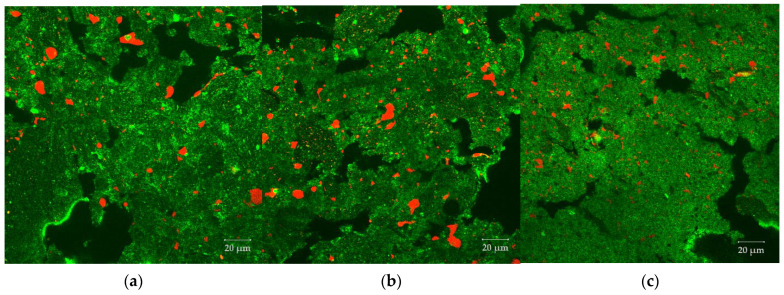
Effect of the screw speed on the morphology of the dispersed oil phase (red) in the protein matrix (green). CLSM images of samples extruded at different screw speeds (**a**) 200 rpm, (**b**) 400 rpm and (**c**) 800 rpm. The barrel temperature was 120 °C and oil was added at position 7.

Figure 11 shows CLSM images of samples with an oil content of 4% extruded at three different screw speeds. For these samples, the oil was added at position 7. From (a) to (c), as the screw speed increases (200 to 800 rpm), the size of the oil droplets decreases. The results of the cumulative droplet size distributions confirm that the droplet area decreases with increasing screw speed. An increase in screw speed leads to higher mechanical stresses, which is expected to enhance the droplet breakup [52,53]. In addition, the higher energy input accelerates the polymerization of the gluten in the screw section, which increases the viscosity and thus again the mechanical stresses applied to the oil droplets [33].

## 4. Conclusions

The addition of oil in the extrusion of wheat gluten with high moisture content led to a considerable change in the process conditions, rheological properties, and product characteristics. As the oil content increased, the material temperature and die pressure decreased. This was attributed to the reduced thermomechanical energy input due to the decrease in material viscosity as well as the possible lubricating effect of the oil. The results suggest that the rate of polymerization decreased with lower thermomechanical treatment, and thus the effect of oil on the material viscosity was amplified along the extruder when oil was added at the front of the extruder. From the images of the teared extrudate samples as well as the micro-CT images, it was evident that the anisotropic product structure was less pronounced with increasing oil content. By adding oil at the end of the extruder, the anisotropic structure of the samples was more pronounced. Moreover, the Young’s modulus of the extrudates decreased with increasing oil content, suggesting an *amplified oil effect* on dynamic processing conditions and protein polymerization. This effect was considerably reduced by adding oil at the end of the extruder, which could be used as a possible strategy in further extrusion applications involving oil addition. CLSM images of the extruded samples revealed that the oil phase was dispersed into small, irregular droplets within the gluten matrix. It was found that the size of the oil droplets was also affected by the oil content and the oil addition point. As the oil content increased, the oil droplet size increased, indicating the occurrence of coalescence. Furthermore, when the oil was added at the end of the extruder, smaller oil droplets were obtained. This is consistent with the expectation that oil droplet breakup is enhanced at higher matrix viscosities.

Overall, the results suggest that oil addition affects the process dynamics at different levels and that better control of this process can only be achieved through a better understanding of the effects of oil on process conditions, protein polymerization, rheological properties of the matrix, and its microstructure.

## Figures and Tables

**Table 1:** Images of the extrudate samples teared immediately after extrusion processing. Influence of oil content on the anisotropic structure as a function of the oil addition point (position 2 and 7). The barrel temperature was 120 °C, screw speed was 400 rpm, total mass flow was 10 kg/h and water content was 51–53% for all samples.

Addition Point	Oil Content		
	0%	2%	4%
Position 2	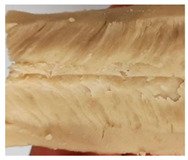	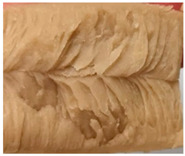	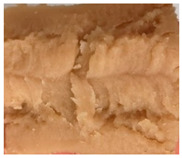
Position 7		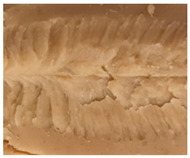	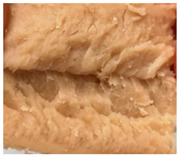

**Table 2:** Micro-computed tomograph (micro-CT) images of freeze-dried extrudate samples. Influence of oil content on the anisotropic structure as a function of the oil addition point (position 2 and 7). The barrel temperature was 120 °C, screw speed was 400 rpm, total mass flow was 10 kg/h and water content was 51–53% for all samples.

Addition Point	Oil Content		
	0%	2%	4%
Position 2	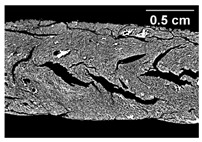	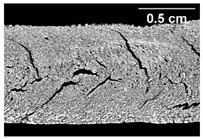	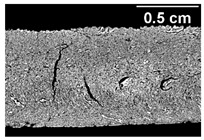
Position 7		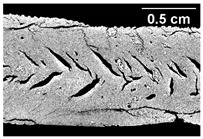	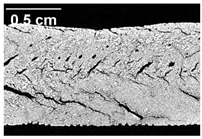

**Table 3:** Images of the extrudate samples teared immediately after extrusion processing. Effect of screw speed on the anisotropic structure of samples with 0 and 4% oil. Oil was added at position 2, barrel temperature was 140 °C, total mass flow was 10 kg/h and water content was 51–53% for all samples.

Oil Content	Screw Speed		
	200 rpm	400 rpm	800 rpm
0%	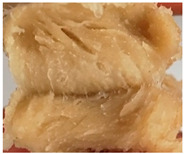	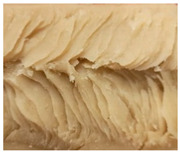	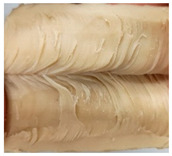
4%	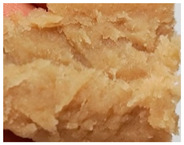	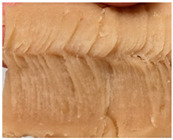	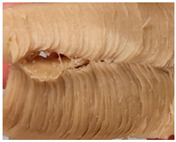

**Table 4:** Micro-CT images of freeze-dried extrudate samples. Effect of screw speed on the anisotropic structure of samples with 0 and 4% oil. Oil was added at position 2, barrel temperature was 140 °C, total mass flow was 10 kg/h and water content was 51–53% for all samples.

Oil Content	Screw Speed		
	200 rpm	400 rpm	800 rpm
0%	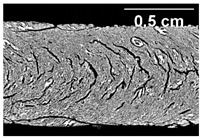	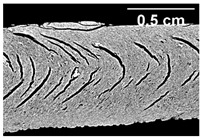	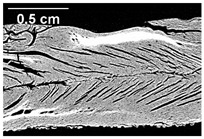
4%	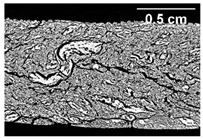	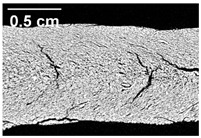	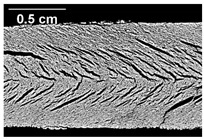

**Table 5:** Influence of oil content on the mean Young’s modulus for samples with oil addition at position 2 and at position 7. The barrel temperature was 120 °C and screw speed was 400 rpm.

Oil Content/%	Young’s Modulus/MPa	
	Position 2	Position 7
0	52.0 ± 7.7	52.0 ± 7.7
2	36.4 ± 3.1	56.3 ± 5.3
4	24.7 ± 2.1	51.9 ± 5.4
